# 

**Published:** 1888-07

**Authors:** 


COMPARATIVE PATHOLOGY.

PARASITES OF THE SHAD AND HERRING. Joseph Leidy, M.D., LL.D. ........ 211
COMPARATIVE PATHOLOGY—ECZEMA. William Alston Edgar, F.R.C.V.S. ........ 216
OSTEOLOGY OF PORZANA CAROLINA—CAROLINA RAIL. R. W. Shufeldt, M.D., U. S. A ..... 231
DOMESTICATION AND FUNCTION. C. M. O'Leary, M.D., LL.D ........ 248
COMPARATIVE STUDIES OF MAMMALIAN BLOOD. Henry F. Formad, M.D ........ 254
VETERINARY DEPARTMENT—UNIVERSITY OF PENNSYLVANIA. William Osler, M.D ........ 312
EDITORIAL DEPARTMENT ........ 315
CASE DEPARTMENT ........ 322
CORRESPONDENCE ........ 325
PROCEEDINGS OF VETERINARY SOCIETIES ........ 328
REVIEWS ........ 332
BOOKS AND PAMPHLETS RECEIVED ........ 339

